# The Role of Adenosine A_2A_ Receptor, CYP450s, and PPARs in the Regulation of Vascular Tone

**DOI:** 10.1155/2017/1720920

**Published:** 2017-08-13

**Authors:** Maan T. Khayat, Mohammed A. Nayeem

**Affiliations:** ^1^Department of Pharmaceutical Sciences, School of Pharmacy, West Virginia University, Morgantown, WV 26506, USA; ^2^Department of Pharmaceutical Chemistry, School of Pharmacy, King Abdulaziz University, Jeddah, Saudi Arabia

## Abstract

Adenosine is an endogenous mediator involved in a myriad of physiologic functions, including vascular tone regulation. It is also implicated in some pathologic conditions. Four distinct receptor subtypes mediate the effects of adenosine, such as its role in the regulation of the vascular tone. Vascular tone regulation is a complex and continuous process which involves many mechanisms and mediators that are not fully disclosed. The vascular endothelium plays a pivotal role in regulating blood flow to and from all body organs. Also, the vascular endothelium is not merely a physical barrier; it is a complex tissue with numerous functions. Among adenosine receptors, A_2A_ receptor subtype (A_2A_AR) stands out as the primary receptor responsible for the vasodilatory effects of adenosine. This review focuses on important effectors of the vascular endothelium, including adenosine, adenosine receptors, EETs (epoxyeicosatrienoic acids), HETEs (hydroxyeicosatetraenoic acids), PPARs (peroxisome proliferator-activated receptors), and K_ATP_ channels. Given the impact of vascular tone regulation in cardiovascular physiology and pathophysiology, better understanding of the mechanisms affecting it could have a significant potential for developing therapeutic agents for cardiovascular diseases.

## 1. Introduction

The vascular system of the human is known to be the major pathway for nutrition exchange among cells as well as organs (i.e., it connects the heart with organs and other tissues) [1]. Likewise, the blood vessels are the tree branches: they originate from the heart through the aorta and conduit arteries to fine capillaries. Blood runs in vessels by proper pressure, and therefore any change or deregulation of blood pressure will result in serious diseases [2]. For example, if blood pressure is low as in hypotension, it could result in organ failure (e.g., acute renal failure) [3]. On the other hand, if blood pressure is high as in hypertension, which is one of the main risk factors for cardiovascular disease, it results in serious diseases such as stroke and chronic renal failure [4]. Currently, hypertension affects over 25% of the human adult population globally [5]. Therefore, it is very important to have optimum blood pressure. Over the past decade, research has been focusing on investigating vascular tone regulation.

Physiologically, vascular tone is the contractile tension of a blood vessel relative to its maximum dilation. Vascular tone regulation is one of the complicated mechanisms in the human body. It involves multiple physiological mechanisms and mediators such as the atrial natriuretic peptide, eicosanoids, adrenal steroids, sodium and water excretion, neurologic control, kallikrein-kinin, and renomedullary endothelial systems [6]. The vascular wall is composed of three layers: intima, media, and adventitia [7]. In the intima layer, the vascular endothelium lines all vessels. The best description of the endothelium could be the printed circuit board (PCB), which is composed of wires, capacitors, and resistors that deliver a particular order by the programmer (i.e., it contains all necessary information for specific functions). Likewise, the vascular endothelium contains all important proteins, enzymes, and ion channels that serve in vascular tone regulation. However, the signaling pathways and their involvement in vascular tone are still not fully clear. The aim of this review is to summarize the previous work of our lab for the past twelve years as well as touch on the related work from other laboratories in relation to vascular tone regulation. In particular, we will address the role of the endothelium, cytochrome P450s (CYPs), and peroxisome proliferator-activated receptors (PPARs) in vascular tone regulation employing disease models, genetic manipulation, and pharmacologic intervention.

## 2. The Role of the Endothelium in Vascular Tone Regulation

For a long time, the endothelium was considered an inert cellophane-like membrane, which coated the entire vascular tree, and its primary function was thought to only regulate the blood vessel wall permeability [8] or to be a mechanical barrier [6]. With the advancement of technology and research, the endothelium is currently viewed as a dynamic, heterogeneous, widely spread tissue that has numerous functions involving secretion, synthesis, metabolism, and immunology [8]. In a human adult, the endothelium consists of between 1 and 6 × 10^13^ cells, weighs about 1 kg, and covers a surface area of 3 m^2^. Also, the endothelium makes up the innermost layer, intima, of all vessels but has different structures and composition based on vessel type [9]. Considering the previous facts and figures, the endothelium is described by some as the largest organ [10, 11]. Since it is a disseminated tissue, it interacts with many systems and has an intricate role in “end organ disease of systems,” which include neurologic, renal, hepatic, cardiac, dermatologic, immunologic, and vascular systems [12].

The role of the endothelium in the vasculature is not only merely to serving as a barrier between the circulation and surrounding tissue; it has a critical function in vascular tone regulation by producing some chemical substances (mediators) that affect vascular hemodynamics. These mediators can be divided into two types: (1) vasodilators, such as endothelium-derived relaxing factors (EDRFs), nitric oxide (NO), prostacyclin (PGI_2_) [8], and endothelium-derived hyperpolarizing factors (EDHFs) [13–16], and (2) vasoconstrictors, which include endothelin-1, reactive oxygen species (ROS), platelet-activating factor (PAF) [8], and arachidonic acid (AA) cyclooxygenase-derived metabolites [17].

It is important to understand the functional complexity of the vascular endothelium; any chronic or acute disruption can cause endothelial dysfunction. This explains its involvement in many diseases such as cardiovascular disease, insulin resistance, obesity, and type 2 diabetes [18, 19]. A clear indicator of endothelial dysfunction is disabling the bioavailability of NO. Moreover, one or more of the following characteristics accompany endothelial dysfunction: reduction of endothelium-mediated vasorelaxation, enhanced cell layer permeability, increased production of reactive oxygen species (ROS), excessive oxidative stress, and overexpression of inflammatory genes [20–24].

The vascular endothelium plays a major role in mediating vascular tone. The cellular level, function, and pathogenesis of the vascular endothelium were explored deeply [8, 19, 25–28];  however, the exact signaling mechanism, enzymes, and substrates involved are not totally revealed. Therefore, we summarized the mechanisms and signaling pathways within the endothelium based on the work done at our lab and by others.

## 3. The Role of A_2A_ Adenosine Receptor in Vascular Tone Regulation

Adenosine receptors are involved in many signaling pathways and downstream effects [14, 15, 29–35]. For instance, they modulate and maintain many mechanisms such as cell growth, apoptosis, cell proliferation, angiogenesis, and immune response in addition to their involvement in diseases like asthma, Parkinson's disease, hypertension, and cancer [29, 36–38]. Moreover, adenosine receptors such as A_2A_ have significant effects in neurodegenerative diseases such as Alzheimer's disease [39], schizophrenia [40], and Huntington's disease [41]. However, A_1_AR is implicated in pathological diseases such as Parkinson's disease and Alzheimer's disease [42]. Therefore, it is worthy to discuss the insightful mechanisms of adenosine and its receptors specifically in vascular tone regulation. The scope of this part is to discuss A_2A_AR and A_1_AR in relation to CYPs, high salt, low salt, and soluble epoxide hydrolase (sEH) in vascular tone regulation. Both receptors belong to the G protein coupled receptors (GPCRs) family [43]. A_1_AR and A_3_AR bind to G_i/o_ proteins family, whereas A_2A_AR and A_2B_AR bind with G_s_ protein. The distribution of A_2A_AR and A_1_AR is not the same in the body [43, 44]. For instance, A_2A_AR is highly distributed in the spleen, thymus, leukocytes, blood platelets, heart, lung, blood vessels, and brain [43]. Particularly in the brain, A_2A_AR is highly expressed in the hippocampus [45], which is involved in Alzheimer's disease [46]. In addition, A_2A_AR is present in astrocytes, oligodendrocytes, microglia, and neurons [47, 48]. It is also reported that A_2A_AR is present in dendritic spines and postsynaptic regions of the basal ganglia [49]. However, with the high A_2A_AR expression in the brain, it has limited expression (“restrictive localization”) in the striatum and olfactory bulb [50]. Moreover, A_2A_AR has various actions such as mediating vasodilation and aiding in new blood vessels build-up [51]. On the other hand, A_1_AR is highly expressed in the CNS on neurons of the cortex, hippocampus, and cerebellum in the brain [50]. Adenosine A_1_ receptor also exists in microglia [52], astrocytes [53], and oligodendrocytes [54]. In addition, A_1_AR is found in other parts of the body such as adipose tissue, heart, and kidneys [44]. In the vascular system, A_1_AR mediates the vasoconstrictive effect of adenosine in vascular beds [55, 56].

GPCRs, including A_1_AR and A_2A_AR, are known to form homo-, multi-, and heterodimers [57]. For example, if the same GPCRs bind together, they form a homodimer, whereas if different receptors bind together, they form a heterodimer [58]. If part of the heteromeric complex is activated, it will manipulate the receptor binding properties of the same complex [59]. Similarly, adenosine receptors are reported to form homodimers among each other and heterodimers with other GPCRs [58]. A good example of homodimer formation in ARs is A_1_AR which has been reported in the intact tissue of the cortex in different species [60] and in Purkinje cells of the cerebellum and hippocampal pyramidal neurons [61]. On the other hand, A_2A_AR homodimers were reported in HeLa and HEK-293T cells cotransfected with different A_2A_AR receptor structures [62]. Ciruela et al. illustrated heterodimerization of A_1_AR and A_2A_AR on the cell surface of HEK-293T cells cotransfected with their cDNAs [63]. The activation of A_2A_AR via CGS 21680 on these cells reduces the binding affinity of a selective radioligand to bind to A_1_AR [63]. Also, A_1_AR and A_2A_AR formed heterodimers in rat astrocyte cultures [64]. The existence of A_1_-A_2A_AR heterodimers in the CNS was found to maintain neuronal excitability [58]. This finding is interesting because it indicates that ARs may affect each other's ability to respond to different ligands even in the case of what we label as selective ligands, as in the case of CGS 21680, which is a selective agonist of A_2A_AR. More interestingly, it was found that GABA uptake was inhibited upon activating A_1_AR whereas GABA uptake was increased upon activating A_2A_AR [64]. The data of adenosine receptor dimerization is mostly reported in the CNS; however, the AR oligomerization investigation in the vasculature is still lacking.

To study the mechanism of action of adenosine receptors, we utilized three major strategies: transgenic animal models, special diet, and pharmacological drugs. Genetically modified mice are utilized to discern the physiological importance of adenosine receptors [50]. Thus, most of the reported work in our lab focused on A_2A_AR-null, sEH-null, and eNOS-null mice. In the special feeding diet, high salt (HS) and normal salt (NS) intake were used as external factors to investigate how adenosine's effect on vascular response may change. Lastly, the pharmacological reagents were useful since they mimicked the actions and signaling pathway of adenosine. For example, CCPA is a selective A_1_AR agonist that can be used in humans, rats, and mice [43]. DPCPX, a xanthine analog, is a specific A_1_AR antagonist [65]. However, NECA, which was originally thought to be a selective A_2_AR agonist, is now considered nonselective for ARs after the development of the more selective A_2A_AR agonist CGS 21680 [66] (more examples are available in Table 1).  Taking into account the reports of dimerization of ARs, it would be prudent to rethink how we use and interpret the data from these pharmacologic agents. Combining the previous strategies provided a better understanding of A_2A_AR's role and function in the vasculature.

Adenosine is a nucleoside generated through metabolic stress as well as high cellular activity [67]. Thus, it increases oxygen supply and decreases oxygen consumption [35]. It is mainly formed by the enzyme 5′-nucleotidase CD73, which dephosphorylates adenosine 5′-monophosphate (AMP) into adenosine that is distributed intracellularly and extracellularly. Intracellularly, adenosine is metabolized by adenosine kinase to AMP, whereas, extracellularly, it is degraded by adenosine deaminase to inosine [68–70]. Since adenosine can vasodilate and hyperpolarize the vascular smooth muscle cells (VSMC), it is considered an EDHF [71]. There are four adenosine subfamily receptors in the vascular tissue that bind to adenosine, which are A_1_AR, A_2A_AR, A_2B_AR, and A_3_AR. These receptors are involved in vascular tone regulation via CYP enzymes family which will be discussed in more detail later. Most of the published data so far suggest that the activation of adenosine receptors A_2A_AR and A_2B_AR mediates vasodilation in various vessels [14, 15, 72–75], whereas the activation of A_1_AR and A_3_AR causes vasoconstriction [55, 56]. Moreover, both A_1_AR and A_2A_AR are necessary for maintaining blood pressure (BP) [76].

The inception studies in our lab of adenosine receptors' role in vascular tone started by studying the vasodilation effect of activating A_2A_AR in A_2A_AR^+/+^ (wild type) and A_2A_AR^−/−^ (A_2A_ knockout) of mouse aorta. NECA (adenosine agonist) and CGS 21680 (a selective A_2A_AR agonist) caused increased vasodilation in A_2A_AR^+/+^ mouse aortae, whereas NECA caused vasoconstriction and CGS 21680 caused neither relaxation nor constriction in A_2A_AR^−/−^ mouse aortae (see Table 1 for all pharmacological reagents) [15]. These findings were important since they partly led to better understanding of the vasodilatory effect of A_2A_AR as evident in the lack of vasodilation when CGS 21680, which is a selective A_2A_AR agonist, was used in A_2A_ knockout mice [15]. However, the nonselective nature of NECA meant that, in the absence of A_2A_ receptors in A_2A_AR^−/−^ mice, this compound would activate the other adenosine receptors (A_1_AR, A_2B_AR, and A_3_AR). The vasoconstrictive effect of A_1_AR, along with the finding that the vasoconstrictive Cyp4a's expression is increased in A_2A_AR^−/−^ mice [15], could explain the NECA-mediated vasoconstriction in A_2A_AR^−/−^ mice [15]. Additionally, the data suggested that A_2A_AR-mediated vasodilation is endothelium-dependant since NECA-induced vasodilation was observed only in aortic rings with intact endothelium, whereas endothelium-denuded rings responded by contraction [15]. In contrast, Arsyad and Dobson illustrated that adenosine-induced vasodilation in male Sprague-Dawley rats' thoracic aortic rings was partially endothelium-dependent [77]. This finding is in agreement with other reported studies in different vascular beds such as rat preglomerular microvessels [72, 78], porcine coronary artery wall [79], porcine retinal arterioles [80], rat pial arterioles [81], rat inferior mesenteric artery [82], and human coronary arterioles [83].

**Figure 1:**
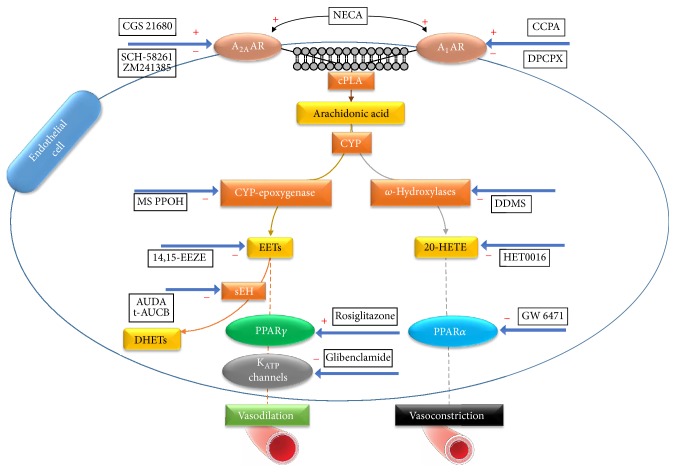
Illustration pathways of A_2A_AR and A_1_AR induced in mice aorta. A_2A_AR induced demonstrates the involvement of CYP-epoxygenase which generates EETs from AA. EETs have substantial involvement in vascular relaxation and they undergo two pathways: (1) they are converted via sEH to DHETs which are inactive or less active metabolites and (2) they activate PPAR*γ* which is involved in other signaling pathways in vascular relaxation. By contrast, A_1_AR induced illustrates the involvement of *ω*-hydroxylases which utilize AA to form 20-HETE. Then, 20-HETE activates PPAR*α* to produce vascular contraction. Different protein targets were probed using pharmacological agonists and antagonists to investigate the possible mechanism and signaling approaches (refer to Table 1 for drugs information). The solid line represents the reported pathways whereas the dashed line shows investigation still underway.

Other studies indicated that the activation of A_2A_AR leads to vascular relaxation via CYP-epoxygenase [15, 75, 84–88]. In these experiments, A_2A_AR^+/+^ and A_2A_AR^−/−^ mice aortae and pharmacological agents such as MS-PPOH (selective CYP-epoxygenase inhibitor), NECA (adenosine agonist), and CGS 21680 (a selective A_2A_AR agonist) were employed. The observed vasodilation induced by NECA and CGS 21680 was completely inhibited by MS-PPOH, which supports the findings we and others have reported in mouse aortae [15], rat preglomerular microvessels [72], and rat isolated perfused kidney [89]. Moreover, the activation of A_2A_AR was associated with upregulation of Cyp2c29 (CYP-epoxygenase enzyme of mice species) in the endothelium-dependent relaxation. In comparison, in A_2A_AR^−/−^ mice, vasoconstriction via Cyp4a (an *ω*-hydroxylase enzyme) was observed [15]. This pathway was explored by using DDMS and HET-0016 (*ω*-hydroxylases inhibitors) (see Figure 1 and Table 1). Both inhibitors reversed vascular contraction, which suggested that the induced vasoconstriction in A_2A_AR^−/−^ mice was mediated by the Cyp4a enzyme [15]. Additionally, the endothelium layer was denuded to investigate its involvement in A_2A_AR-induced vascular dilation. Vasoconstriction was observed in induced A_2A_AR aorta; that is, the CGS 21680 agonist effect on A_2A_AR was abolished in the denuded endothelium [15]. From the previous work and other studies, it is concluded that both CYP-epoxygenases and *ω*-hydroxylases were involved simultaneously with A_2A_AR in vascular tone regulation [15, 90].

A group of metabolites have been linked to the actions mediated by adenosine receptors; studies have demonstrated that the vasodilatory effects induced by adenosine through A_2A_AR and A_2B_AR are mediated by several physiological factors including NO [80, 91], EETs [92], and ATP-sensitive K^+^ (K_ATP_) channels [74, 80, 93, 94]. These studies also used pharmacological reagents, such as glibenclamide, for inhibiting the K_ATP_ channels as well as potassium channels openers (PCO) such as pinacidil and cromakalim. One of the major effects of the opening of K_ATP_ channels in vascular vessels is hyperpolarization of the smooth muscle resulting in vasorelaxation [95, 96]. Moreover, K_ATP_ channels were involved in the signaling pathways of adenosine-induced vasodilation as reported by others in coronary arterioles of miniature swine [97], human isolated small coronary arteries [98], rat retinal microvessels [99], porcine coronary artery [100], isolated rat pulmonary artery rings [101], and perfused hydronephrotic rat kidney [102]. Also, Arsyad and Dobson demonstrated that voltage-dependent K_*v*_ and K_ATP_ are opened in response to A_2A_AR-induced vasodilation in response to adenosine activation [77]. Using PCO in both A_2A_AR^−/−^ and A_2A_AR^+/+^, vasorelaxation was significantly reduced in A_2A_AR^−/−^ compared to A_2A_AR^+/+^, which indicated a role of sarcolemmal K_ATP_ channels with A_2A_AR^+/+^ in vascular relaxation [95]. The PCO-induced effect was inhibited by glibenclamide, confirming the role of PCO in opening the K_ATP_ channels [95]. Therefore, the induced A_2A_AR effect through CGS 21680 was blocked by glibenclamide confirming the involvement of sarcolemmal K_ATP_ channels in the vasorelaxation process [95].

The involvement of A_2A_AR in vascular relaxation through sarcolemmal K_ATP_ channels was investigated. The principle of this study is based on utilizing PCO in A_2B_AR^−/−^ and A_2A/2B_AR^−/−^ mice aortae. The A_2B_AR^−/−^ had a comparable relaxation effect to the A_2B_AR^+/+^ (WT), whereas the A_2A/2B_AR^−/−^ had decreased relaxation compared to A_2B_AR^−/−^. Further blocking A_2A_AR with SCH 58261 (A_2A_AR antagonist) generated the same response as in the A_2A_AR^−/−^. With the comparable effect observed from both A_2A_AR and A_2A_AR^−/−^, it is suggested that the potassium channels mediate the relaxation induced by A_2A_AR.

It is reported by Ye et al. [92, 103] and Lu et al. [104] that EETs act as activators of K_ATP_ channels. Another study by Ponnoth et al. investigated the relationship between EETs and K_ATP_ channels [95]. By blocking the CYP-epoxygenases with MS-PPOH, the relaxation effect induced by pinacidil was greatly decreased. Other laboratories investigated the involvement of NO in A_2A_AR-K_ATP_ pathway in pig coronary arterioles [74], porcine second-order retinal arterioles [80], and mouse aorta [95]. Their studies suggested that A_2A_AR-mediated vasodilation was through NO (released by endothelium) and opening of K_ATP_ channels on smooth muscles [74]. A link in the signaling cascade between the K_ATP_ channels and adenosine-mediated vasorelaxation was reported in several vascular beds [97–102]. In our lab, the K_ATP_ channel openers pinacidil and cromakalim produced relaxation in both wild-type (A_2A_AR^+/+^) and A_2A_AR knockout (A_2A_AR^−/−^) mice but were significantly higher in the presence of A_2A_AR [95]. This finding confirmed that adenosine-mediated vasorelaxation is mediated by K_ATP_ channels [95]. Moreover, we demonstrated that NO played a role in the relaxation induced by pinacidil [95]. When l-NAME (NO synthase inhibitor) was used in wild type (A_2A_AR^+/+^) in the presence of pinacidil, the vascular relaxation was decreased to a level comparable to that in A_2A_AR^−/−^ mice [95]. Based on that, the signaling mechanism of adenosine at the A_2A_AR for aortic vascular relaxation involved CYP-epoxygenases through the following mediators arranged in order: EETs, nitric oxide (NO), and the opening of sarcolemmal K_ATP_ channels in the endothelium [95].

The involvement of 20-HETE with A_1_AR was also investigated in vascular tone of mouse aorta [105]. Since 20-HETE is the active metabolite of AA through Cyp4a and is a potent vasoconstrictor [88], HET0016 was used to inhibit Cyp4a activity. As a result, the vascular contraction of induced A_1_ was notably decreased in A_2A_AR^+/+^ and A_2A_AR^−/−^, suggesting that Cyp4a is downstream of A_1_AR [105].

Other studies were conducted to reveal the involvement of CYP enzymes and signaling pathways in A_2A_AR^+/+^ and A_2A_AR^−/−^ vascular endothelium of mice aortae. First, in A_2A_AR^−/−^ mice, the data demonstrated vasoconstriction induced by Cyp4a through 20-HETE accompanied with upregulation of A_1_AR, which mainly activates protein kinase C (PKC-*α*) and mitogen-activated protein kinase (MAPK) [105]. This effect on PKC is mediated by G*α*i coupling [106]. The involvement of PKC-*α* and MAPK in induced A_1_AR was also determined. In PKC-*α*, using Gö-6976, a selective PKC-*α* inhibitor, it primarily weakens the vasoconstriction response mediated by A_1_AR in both A_2A_AR^+/+^ and A_2A_AR^−/−^ aortae [105]. However, the MAPK inhibitor, PD-98059, was used first to treat aortae, followed by CCPA to activate A_1_AR. PD-98059 totally blocked A_1_AR-induced vasoconstriction by CCPA suggesting the involvement of MAPK in vasoconstriction. Ponnoth et al. [105] and Ansari et al. [107] suggested that A_1_AR-induced vascular contraction is through Cyp4a, which generates 20-HETE, and leads to the activation of PKC-*α*, which phosphorylates MAPK.

We mentioned earlier that the adenosine receptors, like other GPRCs, can interact with each other through dimerization. Ponnoth et al. reported that CCPA (a selective A_1_ agonist) produced significantly higher contraction in A_2A_AR^−/−^ compared to wild-type (A_2A_AR^+/+^) mice [105]. It is also reported by the same authors [105] that the basal protein expression of A_1_AR was higher in A_2A_AR^−/−^ mice compared to A_2A_AR^+/+^ mice [105]. This overexpression is due to lack of A_2A_AR inhibition effect over A_1_AR [105]. The more enhanced contraction in A_2A_AR^−/−^ mice by an A_1_AR agonist could be explained by the increased expression of A_1_AR in this mouse genotype [105] and a possible lack of interaction, as suggested by the reported dimerization, between A_1_AR and A_2A_AR due to the absence of the latter [58, 63, 64].

High salt (HS) intake is considered one of the risk factors for many diseases such as hypertension [108, 109]. HS also acts as an external factor to affect vascular tone, and therefore it is important to study its potential involvement with A_2A_AR and its downstream mechanism in vascular tone regulation, to which we turn our focus now. We previously reported a connection between adenosine receptors activation, particularly A_2A_, and high dietary salt intake in mouse aorta [14, 33, 76] as done by others in isolated perfused rat kidney [89]. Therefore, the role of A_2A_AR in high salt diet was investigated [76]. Data showed upregulation of A_2A_AR expression in response to high salt intake [14, 76, 110] and subsequent increase in A_2A_AR-induced vasorelaxation [14, 33, 72, 89, 110]. In contrast, A_1_AR, which is involved in vasoconstriction, was downregulated in high salt-fed mice [14, 110]. Moreover, the produced adenosine levels were increased by HS diet ingestion in mice [111] and rats in other studies [33, 110, 112, 113].

Another study showed a relationship between activated A_2A_AR and HS diet [14, 16, 78]. The data suggested that HS in A_2A_AR^+/+^ improves A_2A_AR-induced relaxation due to the enhanced CYP-epoxygenases activity, which produce more EETs. On the other hand, in A_2A_AR^−/−^, the HS caused vascular contraction due to the lower level of CYP-epoxygenases activity and enhanced level of A_1_AR [76].

The use of NECA (a nonselective adenosine analog) and CGS 21680 (selective A_2A_AR agonist) resulted in enhanced aortic vasodilation in HS-fed versus NS-fed mice [14]. The close relationship of A_2A_AR either activated or induced via pharmacological drug and HS diet provided a better understanding of vascular modulation. Therefore, we investigated A_2A_AR^+/+^ and A_2A_R^−/−^ mice in HS and normal salt (NS) diet study [76]. In the presence of A_2A_AR, data demonstrated that HS increases A_2A_AR-induced vasodilation. On the other hand, in the knockout mice, HS increases contraction through increased levels of A_1_AR [76]. The HS and NS diet studies confirm the involvement of A_2A_AR in vascular tone regulation. The A_2A_AR response to HS-fed mice is possibly adaptive since it showed upregulation of A_2A_AR and increased vasodilation of mice aortae [76] as previously demonstrated in preglomerular vessels of HS-fed mice [110].

Overall, the many experiments conducted by our lab and others have repeatedly confirmed the important role of adenosine, through its receptor subtypes, in vascular tone regulation. Also, they elucidated much of the downstream signaling pathways, which include CYP enzymes, EETs, *ω*-HETEs, PPARs, and K_ATP_ channels.

## 4. The Role of CYP-Epoxygenase in Vascular Tone Modulation

The investigated downstream mechanism starting from A_2A_AR and A_1_AR until CYP enzymes was discussed above in detail. Now, we will continue the discussion of the other steps in the downstream cascade from CYPs until the hyperpolarization which ultimately leads to vascular smooth muscle relaxation. In this section, we will explore the involvement of CYP enzymes, sEH, PPAR*γ*, and PPAR*α* in vascular tone regulation (Figure 1) under normal and high salt conditions in the aorta and their roles in coronary reactive hyperemia (CRH) in response to ischemia.

A_2A_AR-mediated vascular response involves CYP enzymes, which are related to cytochrome P450s (CYP450) enzyme family [90, 114]. CYP450 family are divided into two types, CYP-epoxygenases (CYP2C and CYP2J) and *ω*-hydroxylases (CYP4A and CYP4F), that are involved in maintaining vasculature tone [90, 114, 115]. Although both enzymes are from the same CYP450 family, they produce different metabolites from AA with widely different physiological functions. For instance, CYP-epoxygenases metabolize AA to generate epoxyeicosatrienoic acids (EETs), which have a vasodilatory effect [116]. On the other hand, the *ω*-hydroxylases convert AA to 20-hydroxyeicosatetraenoic acid (20-HETE), which is a vasoconstrictor [117]. Both groups of metabolites from AA are natriuretic agents.

EETs have four different regioisomers, 5,6-EET, 8,9-EET, 11,12-EET, and 14,15-EET, and have many biological functions; for example, they are released from endothelial cells and act as EDHFs to produce vasodilation through hyperpolarization [118, 119]. Their relaxation of vascular smooth muscle cells is believed to be through the activation of large conductance Ca^2+^-activated K^+^ channels (BK_Ca_) [118, 119]. Moreover, EETs have cardioprotective effects in response to ischemia/reperfusion injury (i.e., EETs reduced heart injury after ischemia) [120]. The vasodilatory effect of EETs is reported in different vascular beds such as the brain [121], preglomerular vasculature of the kidney [122], conduit arteries [14, 15, 32, 33], and intestines [116].

EETs are further metabolized to dihydroxyeicosatrienoic acids (DHETs), which are less active, through soluble epoxide hydrolase (sEH), the primary metabolic pathway for EETs [123]. DHETs were shown to be either inactive or less active than their parent compounds EETs on smooth muscle cells [124]. Other enzyme families involved in AA metabolism are cyclooxygenase (COX) and lipoxygenase (LOX), in which they generate prostanoids and midchain HETEs, respectively. Overall, the metabolites generated by CYPs enzymes from AA are known as oxylipins.

The link between A_2A_AR and CYP-epoxygenase was demonstrated earlier; most collected data so far demonstrated the relationship among A_2A_AR, CYP-epoxygenase, sEH, and peroxisome proliferator-activated receptor gamma (PPAR*γ*) in addition to the involvement of A_1_AR and PPAR*α* in sEH^+/+^ (wild type) and sEH^−/−^ (knockout) in regulating the vascular tone of mice aortae [32]. In sEH^−/−^, the vascular relaxation induced by adenosine was driven by the upregulation of A_2A_AR, CYP2J, and PPAR*γ* and downregulation of A_1_AR and PPAR*α* [32]. However, in sEH^+/+^, adenosine produced vasocontraction through PPAR*α* [32]. Nayeem et al. illustrated that CGS 21680-induced relaxation in sEH^−/−^ mice was induced by A_2A_AR activation, which resulted in CYP-epoxygenases generation of EETs. Moreover, CGS 21680-induced vasodilation was inhibited by the EET antagonist 14,15-EEZE in sEH^−/−^ mice. This finding is in agreement with previous data in which the adenosine-induced vasodilation was blocked by 14,15-EEZE in A_2A_AR^+/+^ mouse aorta [14]. Likewise, Gauthier et al. confirmed the relationship between A_2A_AR and sEH in bovine coronary arteries [125]. Additionally, in sEH^−/−^ mouse aorta, CYP2J5 protein level was upregulated compared to sEH^+/+^, suggesting that the adenosine-induced vasodilation requires CYP-epoxygenases, such as CYP2J [32].

The sEH inhibitors were valuable in linking A_2A_AR with sEH enzyme in vascular tone regulation. Herein, blocking sEH, which converts EETs (vasodilator) to DHETE (inactive) by AUDA or* t*-AUCB, further enhanced the vascular relaxation induced by CGS 21680 [32]. The strong response was suggested to be due to an increase in EET level based on other published data [126]. Moreover, the use of EETs antagonist, 14,15-EEZE, blocked the vascular relaxation induced by CGS 21680, which confirmed that the enhanced vasodilation was due to the increased effect of EET [32].

We evaluated the effect of high salt intake and the role of CYP-epoxygenases in vascular tone regulation [76]. We mentioned above the significance of A_2A_AR and A_1_AR in relation to HS intake in vascular modulation. The role of cyclooxygenase (COX) was investigated by using A_2A_AR^+/+^ and A_2A_AR^−/−^ mice aortae in vascular response. By treating HS-fed A_2A_AR^+/+^ with CGS 21680, in the presence or absence of a COX inhibitor and an eNOS inhibitor, the same vasodilation was produced. This can be explained by the notion that vascular dilation in response to CGS 21680 was COX- and NO-independent [76]. Moreover, the use of EETs antagonist inhibited CGS 21680-induced vasodilation. This illustration is in agreement with previous studies [14, 15, 72, 78]. Additionally, in HS-fed A_2A_AR^+/+^ mice, the level of CYP2c29, which generates EETs, was increased versus NS-fed mice [76]. In comparison with A_2A_AR^−/−^, the expression level of CYP2c29 was reduced in HS-fed versus NS diet A_2A_AR^+/+^. This observation suggests that CYP-epoxygenases, such as cyp2c29, have a substantial role in mediating vascular relaxation downstream of A_2A_AR in response to HS intake. In rat studies of HS-fed A_2A_AR^−/−^ [110], there was no increase of CYP-epoxygenase in response to HS, which supports this finding. Although the findings of Arsyad and Dobson supported our finding that COX was not involved in adenosine-induced vasodilation [77], they contrasted our finding that CGS 21680-induced vasodilation was NO-independent by demonstrating that the adenosine-induced vasodilation in thoracic aortic rings of male Sprague-Dawley rats was NO-dependent [77].

We sought to search for more evidence in our research on vascular tone regulation and moved a step forward from in vitro studies (vessel organ bath) to the ex vivo experiment Langendorff (isolated heart). This extension added more valuable understanding by bringing research data and hypothesis a step closer to translational, functional, and applied science. Herein, we evaluated the role of CYP-epoxygenases and sEH in coronary artery response to ischemia, or coronary reactive hyperemia (CRH), which is a protective mechanism to prevent potential damage to the heart due to ischemic insult [124]. When the heart is exposed to a brief ischemia, coronary blood flow immediately increases afterwards to compensate for the lack of blood supply and avoid any toxic metabolites build-up in the heart [127, 128]. We investigated the pharmacological inhibition on CYP-epoxygenases and sEH using two different genotypes of mice, sEH null (sEH^−/−^) and wild type (sEH^+/+^), using Langendorff (isolated heart) technique [124]. The inhibition of CYP-epoxygenases by MS-PPOH reduced CRH, whereas the inhibition of sEH by* t*-AUCB enhanced CRH in isolated mouse heart [124]. As mentioned previously, CYP-epoxygenases convert AA to EETs. It is reported that EETs were involved in insulin-induced augmentation of skeletal muscle perfusion. The study used MS-PPOH (CYP-epoxygenase inhibitor) to block EETs synthesis and confirmed EETs involvement in the augmentation of skeletal muscle perfusion [78]. Fleming reported that the majority of P450 enzymes are highly distributed in coronary arteries and small arterioles [119]. By blocking CYP-epoxygenases pathway, the synthesis of EETs was inhibited, and the protective CRH was attenuated as well [124]. On the other hand, by treating the heart with* t*-AUCB, CRH was enhanced versus nontreated WT mice [124]. In treating sEH^−/−^ mice compared with nontreated sEH^−/−^ mice by* t*-AUCB, there were no significant differences, which emphasizes the selectivity of sEH inhibitor,* t*-AUCB, in CRH response to brief ischemia [124].

The vasodilatory and cardioprotective roles EETs are reiterated in a number of blood vessels. The release of EETs was also linked to A_2A_AR activation and was shown to be responsive to changes in EETs' generating enzymes (CYP-epoxygenases) and EETs' deactivating enzyme (sEH) [76]. These data further support the notion of finding clinical applications for these beneficial metabolites (EETs) through targeting different enzymes in their pathway.

## 5. The Role of PPAR**γ** in A_2A_AR-Induced Vascular Relaxation

PPARs are one of the downstream targets of EETs. Most of the epoxygenases effects are through PPARs stimulation; that is, PPARs activation produces similar effects to those of EETs and A_2A_AR [129]. For instance, the stimulation of PPARs induces vascular tone regulation, vascular cell proliferation, and cell relocation [130, 131]. Moreover, one of the functions of PPAR*γ* in the endothelial cell as reported by Liu et al. [126] is an anti-inflammatory effect. In diabetic mice study, activation of PPAR*γ* caused endothelium-dependent vasodilation, which was found in nondiabetic mice (+*db*/+*m*) [132]. Moreover, others suggested that PPARs have a role in adipogenesis, insulin sensitivity, and regulation of blood vessels tone [133]. In our sEH^+/+^-sEH^−/−^ mice study, the protein level of PPAR*γ* was upregulated, and the proteins level of PPAR*α* was downregulated in response to CGS 21680 in sEH^−/−^ versus sEH^+/+^ mouse aorta [76, 129]. Therefore, these data suggest that PPAR*γ* is involved in CGS 21680-induced vasodilation in sEH^−/−^, whereas the activation of PPAR*α* causes vasocontraction [76, 129] (Figure 1). It was confirmed that the PPAR*α* agonist (GW 7647) reduced the relaxation of CGS 21680 induced dose-dependently. Additionally, the vascular response induced by CGS 21680 was remarkably blocked by PPAR*γ* antagonist in sEH^−/−^ [32]. Overall, PPAR*γ* in the vascular endothelial cell is activated by EETs to cause vascular relaxation. Since PPAR*γ* is linked with EETs, which in turn are generated by enhanced CYP-epoxygenase activity via A_2A_AR-induced vascular response, we reported the role of PPAR*γ* in HS-fed A_2A_AR^−/−^ and A_2A_AR^+/+^ mice. In addition, we also reported the interplay between A_2A_AR, sEH, EETs, PPAR*γ*, and K_ATP_ in vascular response [129]. In this study, CGS 21680, T0070907, rosiglitazone, AUDA, and glibenclamide were utilized. The data demonstrated in HS-fed A_2A_AR^+/+^ that inhibiting PPAR*γ* led to an increase in CGS 21680-induced vasodilation, which links A_2A_AR with PPAR*γ* [129]. Moreover, PPAR*γ* antagonist attenuated AUDA-induced vascular relaxation response, which confirms the link between EETs (through the sEH inhibitor AUDA) and PPAR*γ*. The K_ATP_ channel blocker also attenuated the vasodilation-induced rosiglitazone; this establishes a connection between PPAR*γ* and K_ATP_. On the other hand, in HS-fed A_2A_AR^−/−^, the HS-induced vascular contraction was attenuated by the sEH inhibitor AUDA [129]. Overall, the vascular response to mediators, such as PPAR*γ* agonist, PPAR*γ*-antagonist, sEH inhibitors, and K_ATP_ channel blocker, was altered in response to HS feeding in both A_2A_AR^−/−^ and A_2A_AR^+/+^. These data indicated that these mediators (EETs, PPAR*γ*, and K_ATP_ channels) are downstream of A_2A_AR [129].

In the isolated mouse heart, the data showed that the effect of CRH was decreased by PPAR*γ* antagonist (T0070907), whereas PPAR*γ* agonist (rosiglitazone) enhanced it [124]. Since the* t*-AUCB-enhanced CRH was reduced by the PPAR*γ* antagonist, T0070907, in WT mouse heart, a link between EETs and PPAR*γ* is more likely [124]. This is an indication that the modulation of CRH involves PPAR*γ* receptors through CYP-epoxygenase-EET pathway [124]. Other studies also reported that PPAR*γ* receptors are induced by EETs [129, 134–136]. Liu et al. suggested that activation of PPAR*γ* increased retention of EETs in endothelial cells and enhanced the anti-inflammatory effect upon using a selective sEH inhibitor [136]. The previously reported data with our finding illustrated that PPAR*γ* activation is downstream of CYP-epoxygenase-EET pathway to mediate CRH.

## 6. Conclusion

The vascular endothelium is a complex tissue with numerous functions and roles that potentially affect all body organs and systems. The main physiologic as well as pathologic role of the vascular endothelium is related to its regulation of blood flow, which is known as vascular tone regulation. The complicated process of vascular tone regulation involves receptors, enzymes, ion channels, and many mediators that have not been fully disclosed. Therefore, understanding the vascular tone modulation by different pharmacological reagents and polymorphic or allelic variant models is very significant. Particularly in mice aortae, activation of A_2A_AR triggers multiple steps to elicit a vascular response. A_2A_AR activation is associated with enhanced CYP-epoxygenases activity, which generate EETs from AA, and opening of sarcolemmal K_ATP_ channels. The reported data suggest that CYP-epoxygenases, EETs, PPAR*γ*, and sarcolemmal K_ATP_ channels are involved in vascular tone regulation. By thoroughly understanding the mechanisms controlling vascular tone regulation, we will likely become closer to finding potential targets for pharmacologic intervention for treating pathologies affected by or involving the vascular endothelium.

## Figures and Tables

**Table 1:** List of pharmacological drugs commonly used in vascular response investigation.

Class	Reagents	Reagents' full description
Nonspecific AR agonist	NECA	5′-*N*-Ethylcarboxamidoadenosine

Specific A_2A_AR agonist	CGS 21680	2-*p*-(2-Carboxyethyl)phenethylamino-5′-N-ethylcarboxamidoadenosine hydrochloride hydrate

A_2A_AR antagonist	ZM241385	4-(2-[7-Amino-2-(2-furyl)[1,2,4]triazolo[2,3-*a*][1,3,5]triazin-5-ylamino]ethyl)phenol
	SCH-58261	7-(2-Phenylethyl)-5-amino-2-(2-furyl)-pyrazolo-[4,3-*e*]-1,2,4-triazolo[1,5-*c*]pyrimidine

A_1_AR agonist	CCPA	2-Chloro-N6-cyclopentyladenosine

A_1_AR antagonist	DPCPX	8-Cyclopentyl-1,3-dipropylxanthine

eNOSi	L-NAME	N-Nitroarginine methyl ester

CYP-epoxygenases inhibitor	MS PPOH	N-(Methylsulfonyl)-2-(2-propynyloxy)-benzenehexanamide

CYP-hydroxylase	DDMS	Dibromododecenyl methylsulfimide

Cyclooxygenase inhibitor	Indomethacin	

sEH inhibitors	AUDA	12-(3-adamantan-1-yl-ureido)-dodecanoic acid
	t-AUCB	*trans*-4-[4-(3-Adamantan-1-yl-ureido)-cyclohexyloxy]-benzoic acid

EET antagonist	14,15-EEZE	14,15-Epoxyeicosa-5(*z*)-enoic acid

20-HETE inhibitor	HET0016	N-Hydroxy-N′-(4-*n*-butyl-2-methylphenyl)formamidine

K_ATP_ channel blocker	Glibenclamide	

Mitochondrial-K_ATP_ channel blocker	5-HD	5-Hydroxydecanoate

PPAR*γ* agonist	Rosiglitazone	

PPAR*α* antagonist	GW 6471	N-((2S)-2-(((1Z)-1-Methyl-3-oxo-3-(4-(trifluoromethyl)phenyl)prop-1-enyl)amino)-3-(4-(2-(5-methyl-2-phenyl-1,3-oxazol-4-yl)ethoxy)phenyl)propyl)propanamide
